# Supplementary material for: Anti-Inflammatory Properties of Bellevalia saviczii Root Extract and Its Isolated Homoisoflavonoid (Dracol) Are Mediated by Modification on Calcium Signaling
Source: Molecules. 2019 Sep 17;24(18):3376. doi: 10.3390/molecules24183376 (PMC6766996; doi:10.3390/molecules24183376)
Supplement: Supplementary file 1 [file molecules-24-03376-s001.pdf]

Supplementary Material 1 (S1):

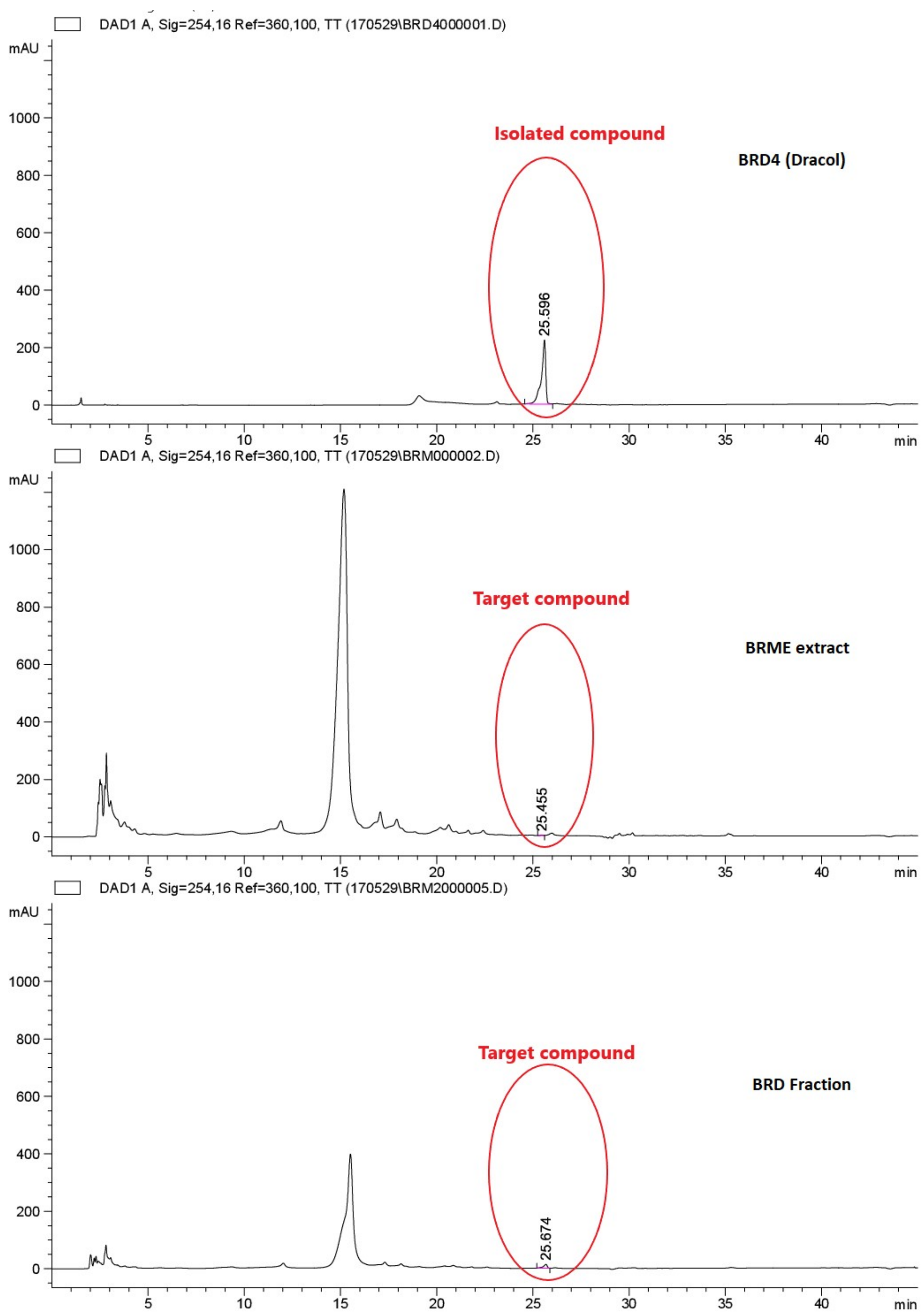

HPLC Fractionation and Isolation of homoisoflavonoid (*Dracol*) isolated from *B*. *saviczii* roots.

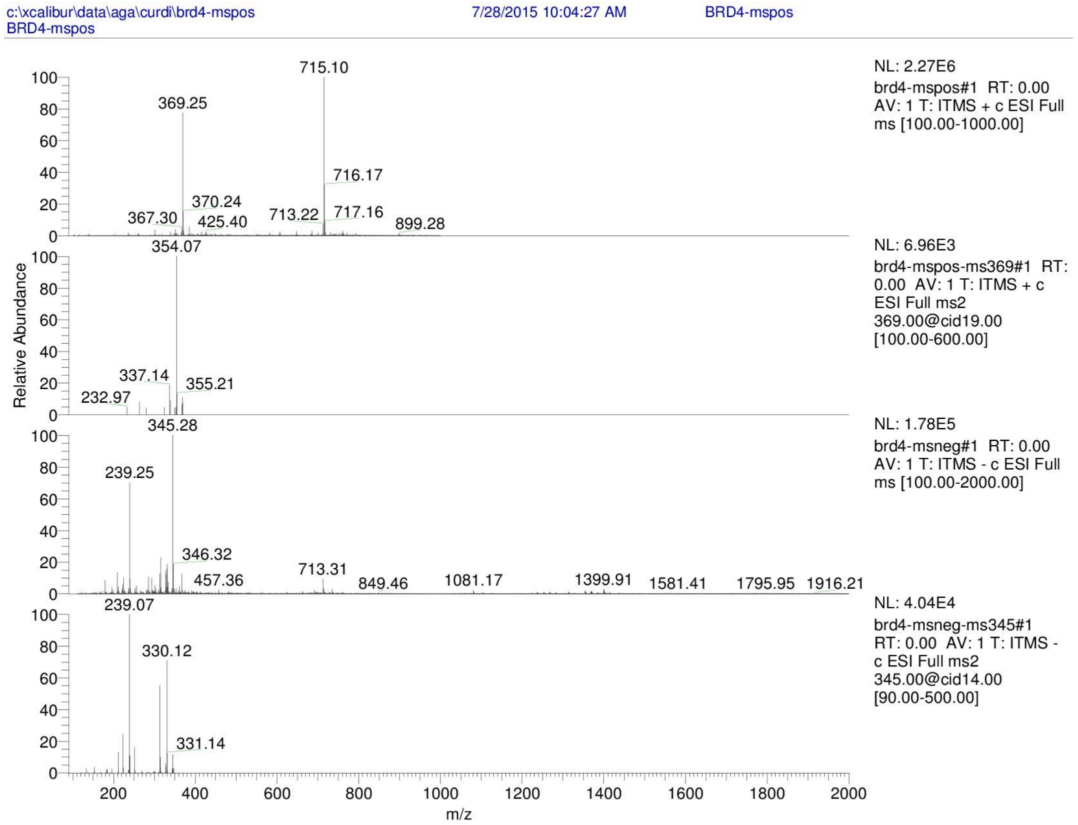

Mass spectra of homoisoflavonoid (*Dracol*) isolated from *B*. *saviczii* roots.

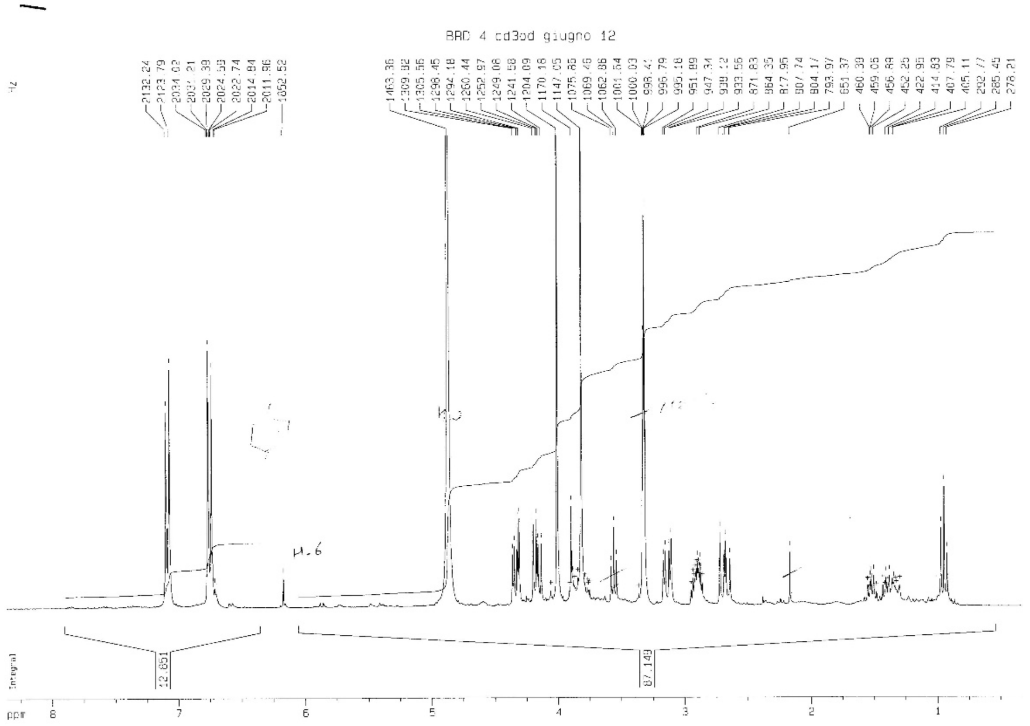

^{1}HNMR spectrum of homoisoflavonoid (*Dracol*) isolated from *B*. *saviczii* roots.

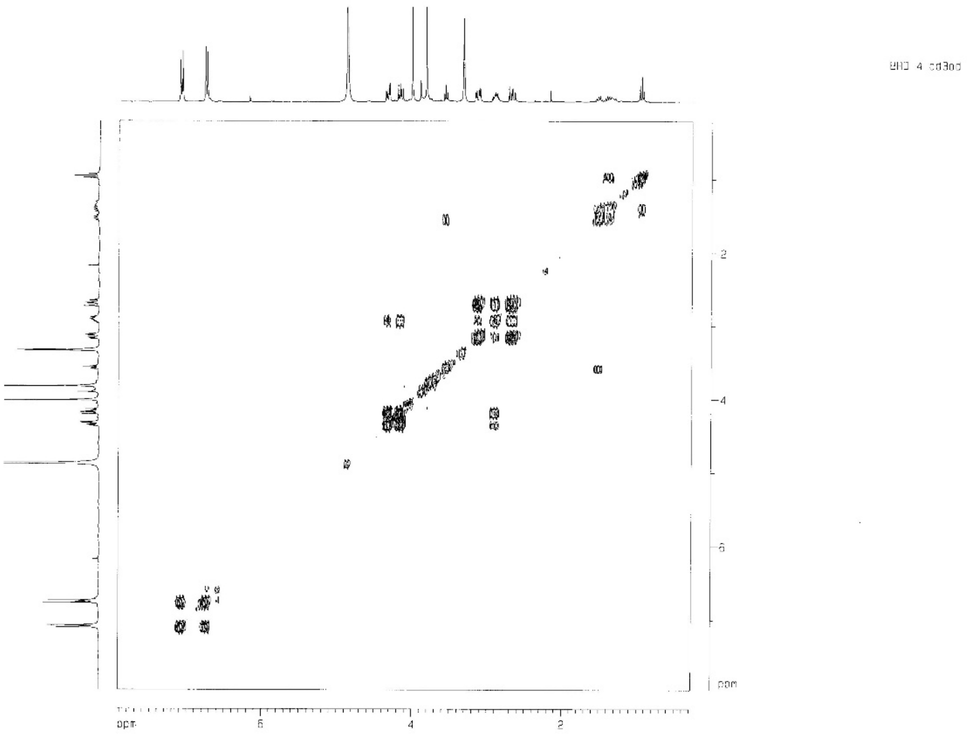

^{1}HNMR-COSYspectrum of homoisoflavonoid (*Dracol*) isolated from *B*. *saviczii* roots.

NF-kB activation with *Dracol* and BRME

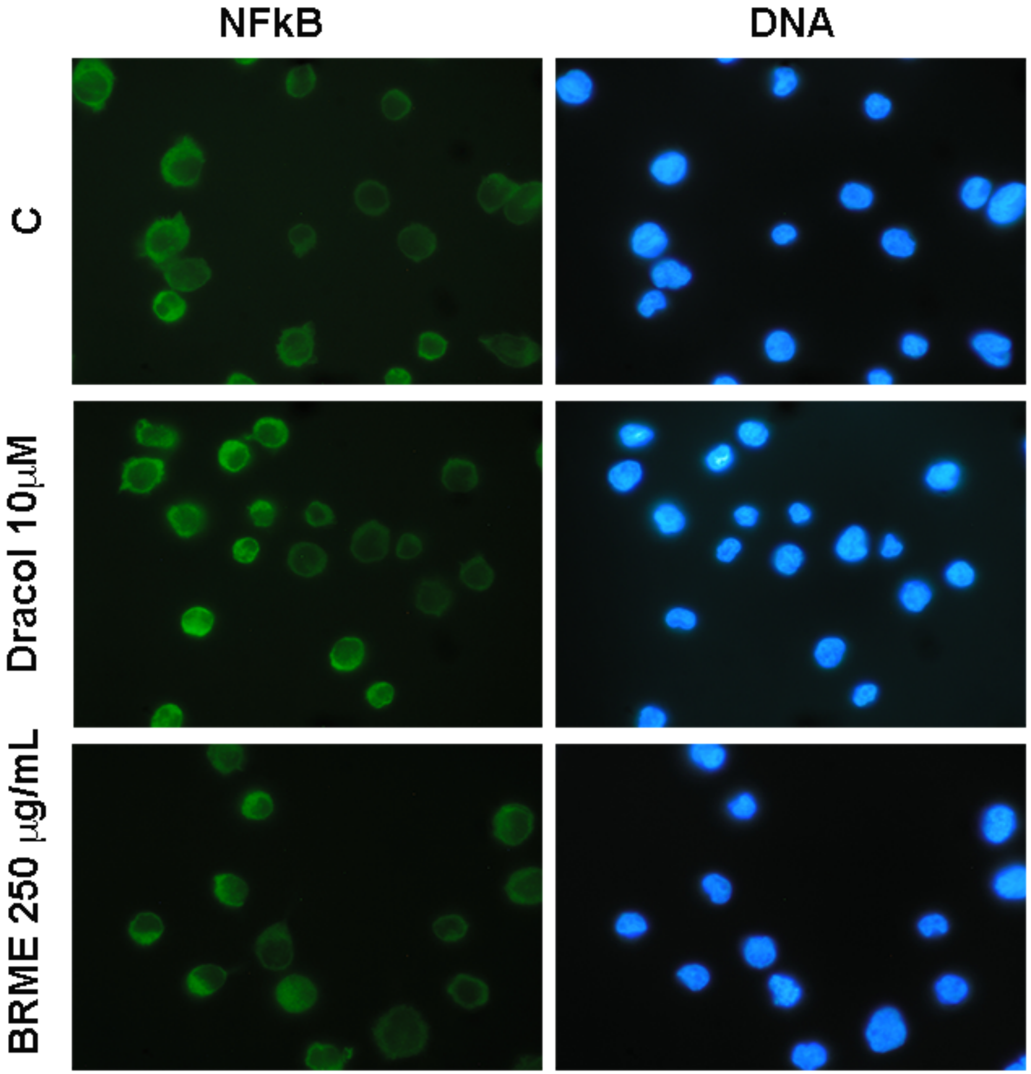

Representative imagines of NF-kB activation with *Dracol* and BRME alone.

NF-kB activation with *Dracol* and BRME

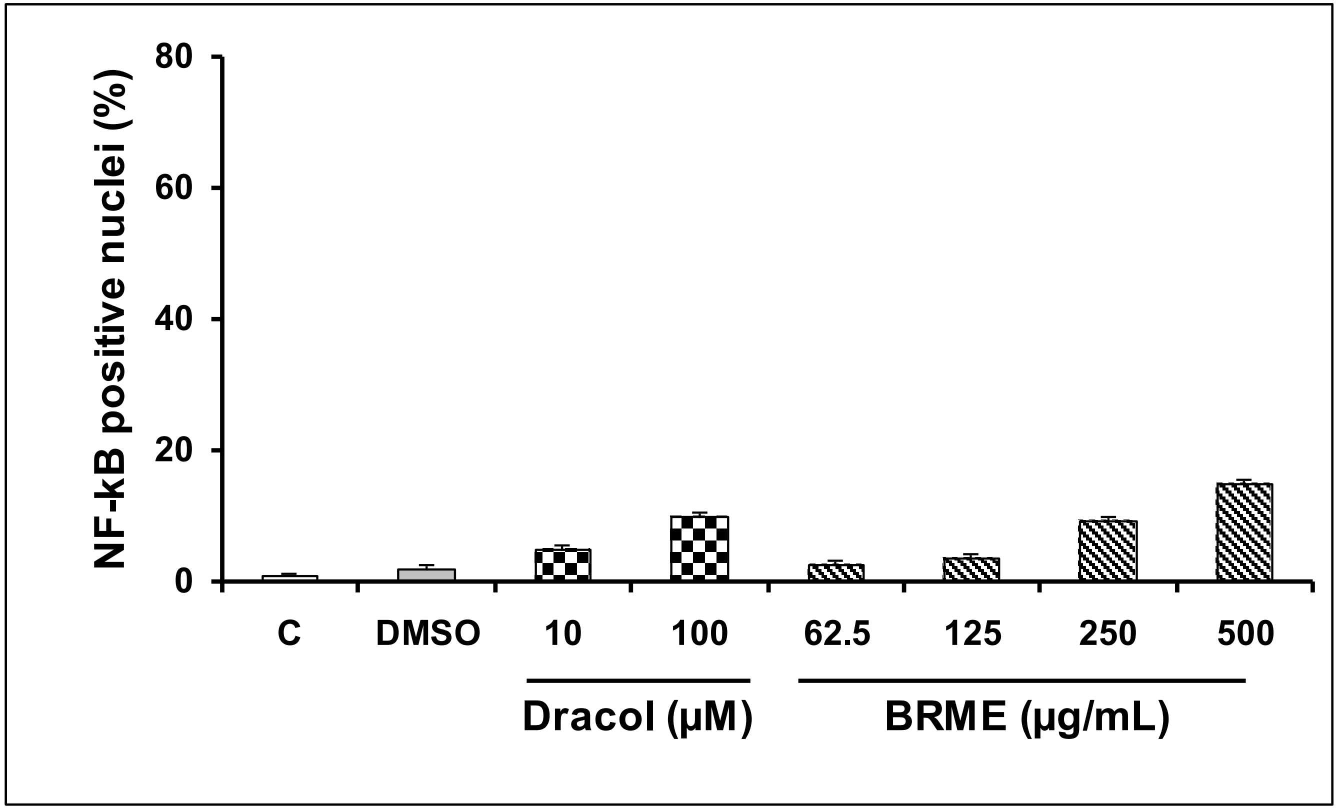

Quantitative analysis of NF-kB activation with *Dracol* and BRME alone obtained by immunofluorescence microscopy. Values referred to NFkB activation are low and comparable to control and DMSO samples.

Representative image of Western Blot of NF-kB activation with Dracol and BRME alone and relative quantitative analysis

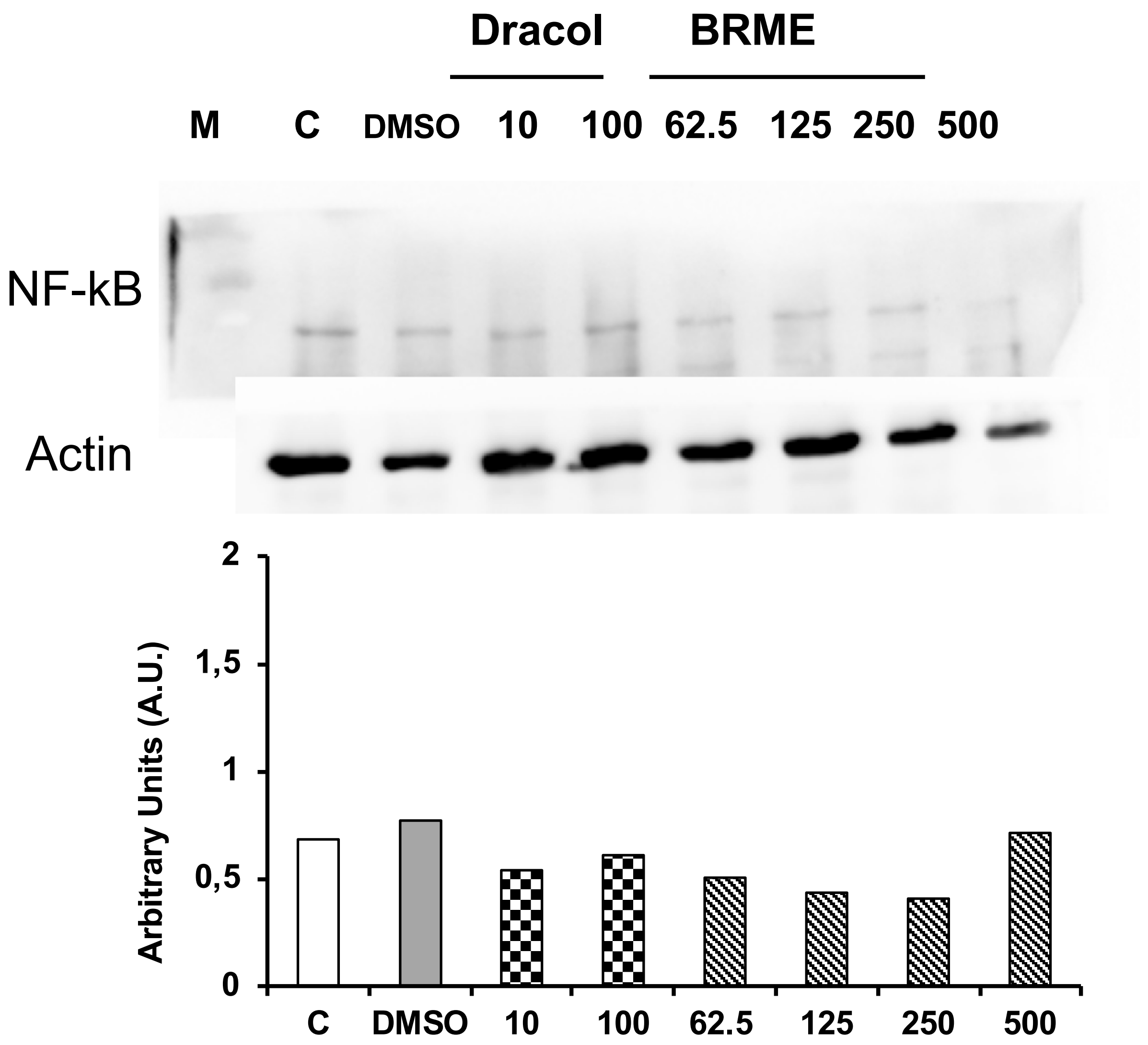

NF-kB values are low and comparable to control and DMSO samples.

TNFα levels with *Dracol* and BRME

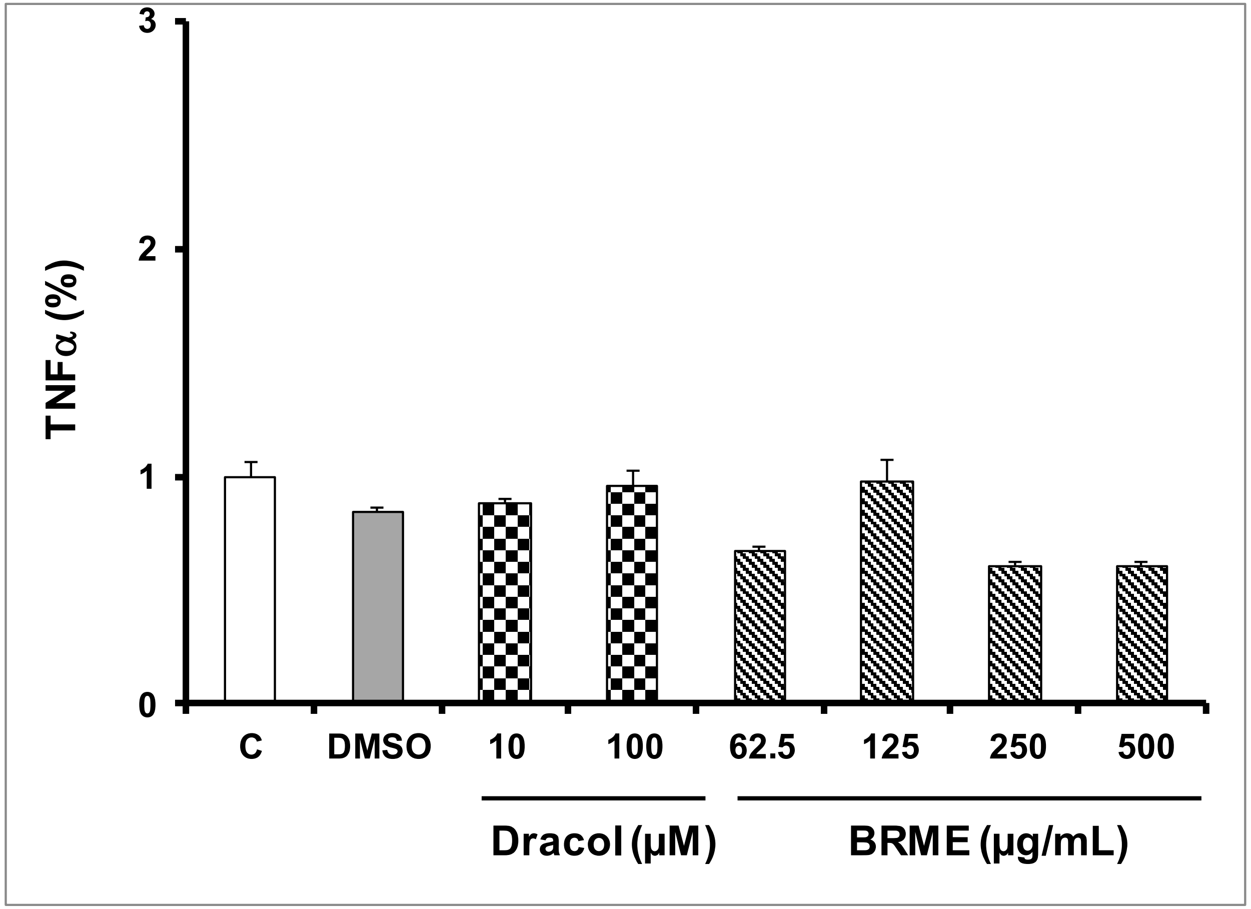

TNFα levels released by THP1, evaluated by ELISA, after treatment with *Dracol* and BRME alone. TNFα values are low and comparable to control and DMSO samples.

IL6 levels with *Dracol* and BRME

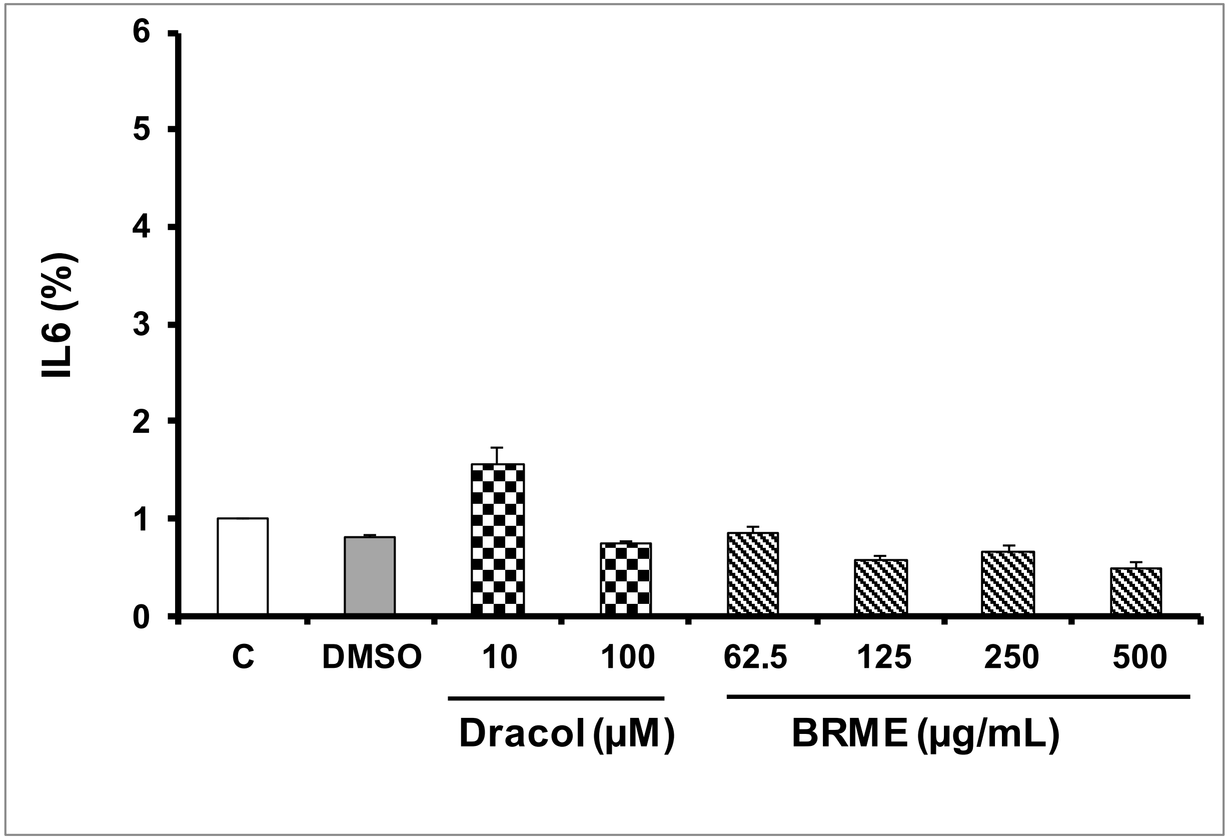

IL6 levels released by THP1, evaluated by ELISA, after treatment with *Dracol* and BRME alone. IL6 values are low and comparable to control and DMSO samples.

IL1β levels with *Dracol* and BRME alone

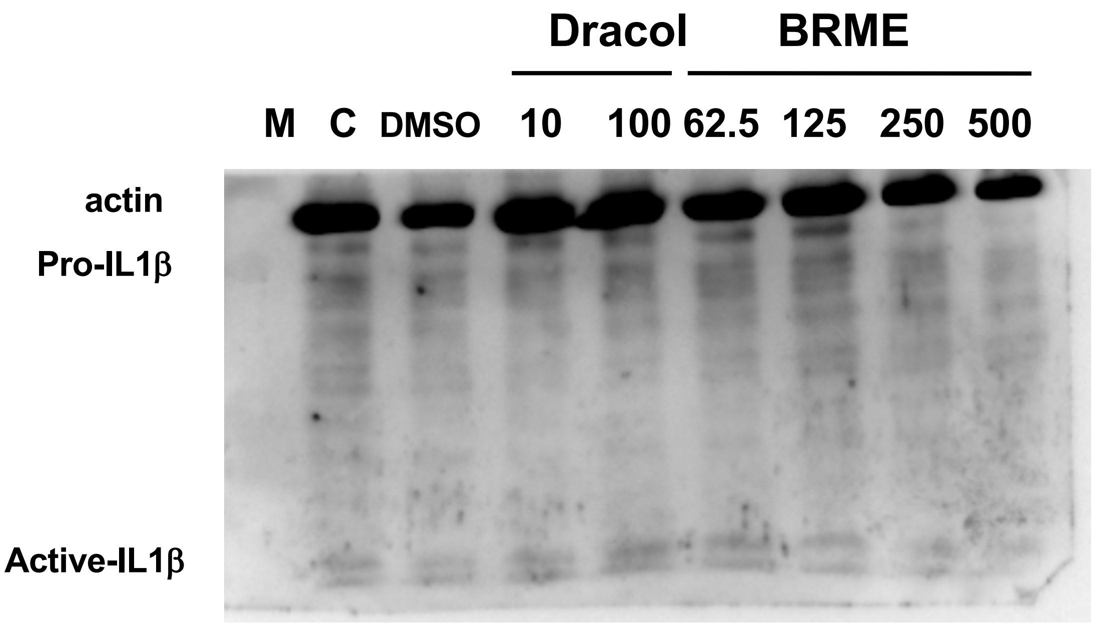

As expected, the absence of LPS did not induce IL1β expression.

THP1 death rate (%) evaluated by Trypan blue exclusion test after treatment

with 200 µM *Dracol* and 1000 µg/mL BRME alone for different days

Trypan Blue (%)	24 h	48 h	72 h	96 h
C	2	3	3	4
Dracol (200 µM)	7	5	7	8
BRME (1000 µg/mL)	6	10	5	5

Supplementary material 3 (S3):

BAPTA reduced LPS-induced p65 NF-kB nuclear translocation

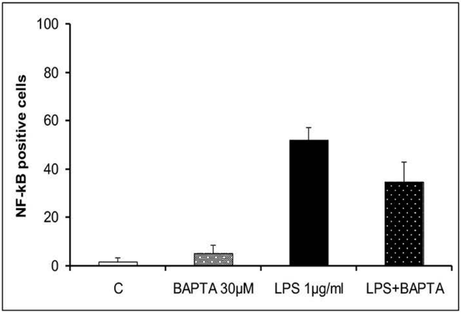

Pre-incubating THP-1 cells with BAPTA (a membrane-permeable buffer of intracellular Ca^{2+}) attenuated p65 NF-kB nuclear translocation in the presence of LPS, thereby confirming the requirement for Ca^{2+} oscillations in this process.

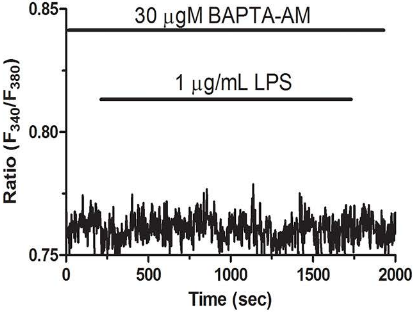

Ca^{2+} oscillations in the presence of BAPTA and LPS
